# Practice at St. Luke’s Hospital

**Published:** 1873-11

**Authors:** 


The Practice at St. Luke's Hospital, New York.—Sir:—In your publication of June 16th appeared a report of "Practice and Peculiarities of Treatment at St. Luke's Hospital," in which there were some inaccuracies, which you will perhaps permit me to correct here, as I was Resident Physician of St. Luke's at the time, though not at all responsible for the article.

I will take them seriatim. In acute rheumatism, the mode of administration of iodide of potassium and sulphate of quinine, was combining them at the time of administration, and not giving them at intervals, as stated.

In order to do this, a solution was made of ten grains to the drachm of water, of either salt, and a fluid drachm of the first and a fluid drachm and a half of the second were added to a third of a tumbler of water. This was given every four hours, and continued without producing cinchonism, for a week in some more obstinate cases. Usually forty-eight or seventy-two hours ended the malady.

The remedies spoken of as efficacious in night-sweats were used with varying results. In most cases a change from one to another accomplished an abatement for a few nights, and it followed whether the change was in one direction or another.

Ergot acted well, perhaps longer than acid, oxide of zinc, hyosciamus or any of the other various remedies.

Sponging the body at evening was grateful to the patient, and a good prophylactic.

In "reduction of high temperature," ice-water was repeatedly used and with most gratifying results, whether employed in acute rheumatism, typhoid fever, or other cases. Life was unquestionably saved by its use in a number of cases when the temperature had reached 106° or 107°, and the patients were comatose.

In one instance, ice was applied to affected joints, in acute articular rheumatism, with recovery in thirty-six hours as a result.

In sub-acute pleurisy the treatment was not at all as uniform as stated, and it was claimed (but not conceded) by each advocate that his treatment was the successful one, whether diurectics and blisters, tonics and no bitters, or paracentesis thoracis was practised. One point was established, that in a case with large effusion which did not subside spontaneously, or during the course of other treatment, if a portion of the fluid were removed by trocar or aspirator, the absorption of the remainder was facilitated, and in but one case did death follow tapping, and here the pleural sac was filled for several weeks while other remedies were employed, and when at last resorted to, the patient was quite exhausted, and only a small amount was taken away.

The statement that in "Bright's disease the general principles embrace the administration of tonics and diuretics," is only true of chronic cases.

The treatment of acute nephritis was most gratifying where milk-diet was adhered to, and no medicine used. The custom was to give the patient whatever amount of milk he or she would take at meals, with cracker or oatmeal or other light matter. At the intervals they drank a cup of milk once in two hours, if they wished it. An increased amount of urine of lower specific gravity and subsidence of the œdema followed, with such promptness as to surprise the practitioner new to the use of it.

